# Dynamic PET Measures of Tau Accumulation in Cognitively Normal Older Adults and Alzheimer’s Disease Patients Measured Using [18F] THK-5351

**DOI:** 10.1371/journal.pone.0158460

**Published:** 2016-06-29

**Authors:** Samuel N. Lockhart, Suzanne L. Baker, Nobuyuki Okamura, Katsutoshi Furukawa, Aiko Ishiki, Shozo Furumoto, Manabu Tashiro, Kazuhiko Yanai, Hiroyuki Arai, Yukitsuka Kudo, Ryuichi Harada, Naoki Tomita, Kotaro Hiraoka, Shoichi Watanuki, William J. Jagust

**Affiliations:** 1 Helen Wills Neuroscience Institute, University of California, Berkeley, California, United States of America; 2 Center of Functional Imaging, Lawrence Berkeley National Laboratory, Berkeley, California, United States of America; 3 Department of Pharmacology, Tohoku University School of Medicine, Sendai, Japan; 4 Institute of Development, Aging and Cancer, Tohoku University, Sendai, Japan; 5 Cyclotron and Radioisotope Center, Tohoku University, Sendai, Japan; University of Manchester, UNITED KINGDOM

## Abstract

**Background:**

[^18^F]THK5351, a recently-developed positron emission tomography (PET) tracer for measuring tau neurofibrillary tangle accumulation, may help researchers examine aging, disease, and tau pathology in living human brains. We examined THK5351 tracer pharmacokinetics to define an optimal acquisition time for static late images.

**Methods:**

Primary measurements were calculation of regional values of distribution volume ratios (DVR) and standardized uptake value ratios (SUVR) in 6 healthy older control and 10 Alzheimer’s disease (AD) participants. We examined associations between DVR and SUVR, searching for a 20 min SUVR time window that was stable and comparable to DVR. We additionally examined diagnostic group differences in this 20 min SUVR.

**Results:**

In healthy controls, [^18^F]THK5351 uptake was low, with increased temporal relative to frontal binding. In AD, regional uptake was substantially higher than in healthy controls, with temporal exceeding frontal binding. Retention in cerebellar gray matter, which was used as the reference region, was low compared to other regions. Both DVR and SUVR values showed minimal change over time after 40 min. SUVR 20–40, 30–50, and 40–60 min were most consistently correlated with DVR; SUVR 40–60 min, the most stable time window, was used in further analyses. Significant (AD > healthy control) group differences existed in temporoparietal regions, with marginal medial temporal differences. We found high basal ganglia SUVR 40–60 min signal, with no group differences.

**Conclusions:**

We examined THK5351, a new PET tracer for measuring tau accumulation, and compared multiple analysis methods for quantifying regional tracer uptake. SUVR 40–60 min performed optimally when examining 20 min SUVR windows, and appears to be a practical method for quantifying relative regional tracer retention. The results of this study offer clinical potential, given the usefulness of THK5351-PET as a biomarker of tau pathology in aging and disease.

## Introduction

Tau pathology is frequently present in the brains of older adults, not only in the presence of Alzheimer’s Disease (AD) [1], but also among cognitively normal older adults (potentially as primary age-related tauopathy or PART) [2]. In particular, neurofibrillary tangles (paired helical filaments), which are comprised of hyperphosphorylated tau protein [3], are a key pathological hallmark of and potential clinical target in AD and other tauopathies [4, 5]. Until recently, efforts to understand late-life pathological tau accumulation have relied upon post-mortem autopsy or pre-mortem collection of cerebrospinal fluid for characterization of tau measures [6–8]. While autopsy measures have obvious limitations, cerebrospinal fluid also does not provide information on the location of tau pathology in the brain, which might be crucially important in interpreting results.

There has recently been substantial progress in the development of radioligand tracers for studying the accumulation of tau pathology in the living human brain with positron emission tomography (PET) [9–10]. These developments come on the heels of major breakthroughs in our ability to image aggregated proteins associated with human disease; β-amyloid imaging was the first example of this. These tau radiotracers include FDDNP [11], T807/AV-1451 [12], and PBB3 [13]. [^18^F]THK5351, one such tracer for *in vivo* tau neurofibrillary tangle accumulation, is a recent molecule in a series of compounds developed at Tohoku University [14]. In the current study, we examined the pharmacokinetics and patterns of THK5351 uptake, and putative tau accumulation, in AD participants and in healthy control (HC) participants, to provide a more thorough understanding of this tracer, and potential processing and analysis considerations for any future researchers using this or related tracers.

Development of any PET radiopharmaceutical imposes a number of requirements related to brain uptake, affinity, selectivity, metabolism, and target to background binding [10]. A particularly useful characteristic for human studies is a pharmacokinetic profile that achieves steady state (i.e., when change in signal over time relative to a reference region approaches and remains near zero) within a reasonable time for scanning, and thus permits use of tissue ratios such as standardized uptake value ratios (SUVRs) for measurement of tracer distribution [15]. Another approach to image quantification is the use of dynamic acquisition of PET data and analysis with graphical methods such as Logan plots (for reversibly bound tracers), producing distribution volume ratios (DVRs) or measures of nondisplaceable binding potential (BP_ND_) [16–17]. Relative to these dynamic measures that rely upon continuous data collection from time of injection, SUVR uses a shorter scan duration, and therefore allows for a wider population to be scanned since limitations on patient tolerance and scanner time affect practical utility [18]. SUVR method disadvantages are overestimation relative to DVR, potentially in a time-varying fashion [19].

We performed this study in order to investigate the temporal dynamics of the tau imaging agent [^18^F]THK5351. The goal of the study was to investigate relationships between dynamic PET data with DVR analyses, and data collected in static modes for SUVR analyses. Specifically, we sought the best time window in which to assess SUVR data. We hypothesized that an optimal THK5351 SUVR time window should result in a high correlation with more dynamic measures (such as DVR) and should permit detection of group differences (AD vs. controls) in regions shown in previous research to have elevated tau pathology in AD [4]. It is also important to assess, for both DVR and SUVR methods, how stable measures are across time, and whether they reach steady state. In the current study, we examined THK5351 SUVRs across multiple regions and time windows, for the optimal properties of low bias, high correlation, and diagnostic group differences. Such findings could not only inform the understanding of the mechanisms of tau pathology in normal aging and neurodegenerative disease, but also could contribute greatly to the body of empirical and theoretical research on tau PET imaging tracers, particularly regarding the kinetics of THK5351, a new and promising tracer. Further, the results of this study offer clinical potential, given the usefulness of THK5351-PET as a biomarker of tau pathology in aging and AD.

## Materials and Methods

The Institutional Review Board of Tohoku University approved the study, and all procedures performed were in accordance with the ethical standards of the Declaration of Helsinki. After complete description of the study to the patients and controls, written informed consent was obtained from the participants or their guardians.

### Overview

We explored tissue binding for this tracer with multiple measures. The primary measurements were calculations of regional DVR values from Logan graphical analysis (using Matlab scripts developed in our laboratory) and SUVR (using FSL image calculation tools; www.fmrib.ox.ac.uk/fsl); no arterial blood sampling was performed. We sought to describe associations between DVR and SUVR, searching for a 20 min SUVR time window that was both stable and comparable to DVR, additionally examining diagnostic group differences in this optimal 20 min SUVR. Also, 4 AD participants were unable to complete the final 30 min of a 90 min scan acquisition; we therefore examined within-measure associations between 60 min (*n* = 16 participants) and 90 min data (*n* = 12).

### Participants

This study included 16 participants: 6 cognitively normal older adult controls, and 10 AD patients (Table 1). In addition, all AD patients were considered amyloid-positive based on Pittsburgh compound B (PiB) scans, and all HC subjects were amyloid-negative; analyses of amyloid imaging data and relations to THK5351 retention are beyond the scope of the current manuscript.

**Table 1:** Participant Demographics.

	Healthy Controls (90 min available)	Alzheimer’s Disease		
		All (60 or 90 min available)	*90 min available*	*60 min available*
*n*	6	10	*6*	*4*
Age, M(SD)	71.2 (7.9)	75.1 (9.6)	*72*.*5 (12*.*0)*	*79*.*0 (1*.*2)*‡
MMSE, M(SD)	28.8 (1.3)	18.4 (4.2)*	*20*.*3 (3*.7)*†	*15*.*5 (3*.3)*†
CDR, M(SD)	0 (0)	1.90 (0.88)*	*1*.*67 (0*.82)*	*2*.*25 (0*.96)*
Years Education	12.5 (1.8)	13.1 (2.3)	*13*.*7 (2*.*0)*	*12*.*3 (2*.*9)*

CDR = Clinical dementia rating score. MMSE = Mini-mental status examination score.

* differs from Healthy Controls, *p* < .05.

^†^ marginal AD subgroup difference, *p* = .07.

^‡^ marginally differs from HC, *p* = .09.

### Subject recruitment and image acquisition

Healthy controls were recruited from the local area of Tohoku University using poster advertisements. These volunteers were taking no centrally-acting medications, and had no cognitive impairment or cerebrovascular lesions identified via MRI. AD patients were recruited through the Tohoku University Hospital Dementia Patients Registry. The diagnosis of AD was made according to the National Institute of Neurologic Disorders and Stroke/Alzheimer’s Disease and Related Disorders Association (NINCDS-ADRDA) criteria. All AD patients had the amnestic subtype of Alzheimer’s Disease. Disease duration for the 10 patients ranged 3 to 7 y (5.3 +/- 1.3 y). In addition, as mentioned above, a positive amyloid PiB scan confirmed AD diagnosis, and AD subjects had CDR scores ranging from 1 to 3 (all HC had CDR = 0). PET imaging was performed using an Eminence STARGATE PET scanner (Shimadzu, Kyoto, Japan). After intravenous injection of [^18^F]THK5351 (185 MBq), dynamic PET images were obtained for 90 min or 60 min without arterial blood sampling. MRI was performed on all participants. T1 MR images were obtained using a SIGNA 1.5-Tesla system (General Electric, Milwaukee, WI). A 3D volumetric acquisition of a T1-weighted gradient echo sequence produced a gapless series of thin axial sections using a vascular TOF SPGR sequence (echo time/repetition time, 2.4/50 ms; flip angle, 45°; acquisition matrix, 256 × 256; 1 excitation; field of view, 22 cm; slice thickness, 2.0 mm). Images (structural T1 MRI and dynamic PET data files) were electronically transferred to the University of California Berkeley/Lawrence Berkeley National Laboratory for processing and analysis.

### THK5351 PET processing

PET data were realigned and coregistered and resampled to the participant’s structural MRI using Matlab and SPM8 (www.fil.ion.ucl.ac.uk/spm/). In order to select suitable parameters for DVR calculation, we first calculated and examined binding potential (BP) values from the simplified reference tissue model (SRTM) [20] approach. Using all ROIs described below across all subjects (S1 Table), we obtained an optimal value of k_2_’ = .115 min^-1^. This SRTM k_2_’ value was used in our Logan graphical analysis approach (DVR). SRTM BP results are largely concordant with Logan DVR measures (S1 Fig), and as Logan DVR methods are more commonly used in research and able to be performed by different labs, we report further analyses using DVR.

Custom-made scripts were used for the calculation of DVR from Logan graphical analysis, and for generation of SUVR images, using a gray matter masked cerebellum reference region [16–17] defined using FreeSurfer (described below). Particular time frames selected for use in specific DVR and SUVR analyses are described within their respective sections below.

### MRI processing

T1 MPRAGE structural MRI scans were processed using FreeSurfer version 5.1 (http://surfer.nmr.mgh.harvard.edu/) to delineate anatomical regions of interest (ROI; S1 Table) masks for multiple brain subregions on the MRI (coregistered to the THK5351 PET scan), in each participant’s native space. This was the manner in which we derived the gray matter cerebellum mask used as a reference region for DVR and SUVR calculations. An eroded white matter mask was created by smoothing the binary FreeSurfer white matter mask by an 8mm 3D Gaussian kernel, then thresholding and masking at a white matter probability of *p* > .7.

### Data analysis

#### Tracer measurement stability: 60 min vs. 90 min data

We compared 60 min to 90 min DVRs to allow comparison across all participants and to see how stable measurements were using different lengths of data. Only a subset (6 of 10) of AD participants in the current study completed the full 90 min PET sequence. We used Pearson correlations to analyze the cases with 90 min of data using both 20–90 min and 20–60 min data sets (starting at 20 min, as Logan plots were nonlinear before this point), and examined *r*^*2*^ and *p* values for HC and AD groups separately and combined.

#### Assessing DVR and SUVR stability over time

In order to examine, for both DVR and SUVR methods, how stable the measures are across time, and whether they potentially reach steady state (or minimal to no change over time), we measured signal and change in signal over time for both methods. Using 10 min time windows, we calculated the mean image DVR and SUVR values, per participant, over bilateral FreeSurfer-defined ROIs. We next calculated the difference between sequential 10 min time windows.

#### Assessing relationships between DVR and SUVR over time

We next calculated the correlations between DVR values (20–90 min) and SUVR values over different time frames. We did this in order to determine which 20 min SUVR value was most closely associated with the DVR value. As we were interested in exploring these relations over the full 90 min scan window that was available on a subset of participants, we performed these analyses on *n* = 12 participants with 90 min of data. We then tested and reported the associations between 20 min SUVR value and 20–90 min DVR value for all adults, as well as for HC and AD separately.

#### SUVR 40–60 image generation

SUVR 40–60 min images were created for each participant in native space, and then normalized to the FSL MNI152 2mm space template. Normalization was done using ANTS (http://stnava.github.io/ANTs/) and with the use of a study-specific intermediate template created from the 6 HC and 10 AD participants described in Table 1. This was done in order to minimize overall deformation for each subject on average. Average images across diagnostic groups were made using FSL, to enable visualization of the pattern of uptake within and between groups.

#### Testing of group differences in 40–60 min SUVR

We used FreeSurfer-generated ROIs to extract mean signal from native-space THK5351 SUVR 40–60 min images. These values were then entered into two-sample *t*-tests using Matlab and tested for significant group differences. As the focus of this manuscript was the methodological understanding of this tracer and its behavior, analyses of group differences in tracer uptake were exploratory and primarily served in the interpretation of other findings.

## Results

### Time-activity curves and Logan graphical analysis

**Fig 1:**
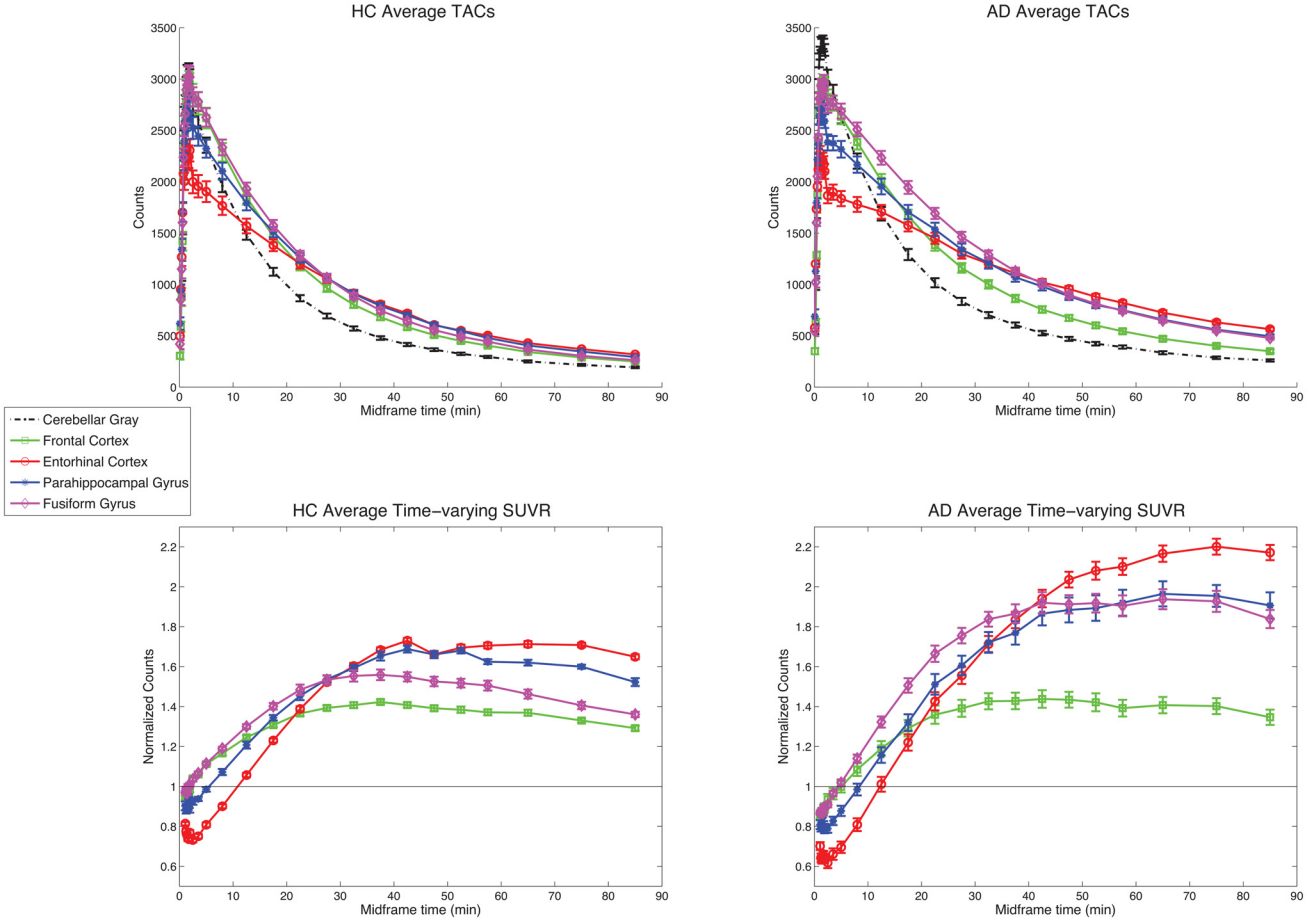
Time-activity curves and time-varying SUVRs. Average time-activity curves (0–90 min) for HC (top left; *n* = 6) and AD (top right; *n* = 6) participants, for 5 select ROIs, and average time-varying SUVRs (1–90 min data shown for clarity) for HC (bottom left; *n* = 6) and AD (bottom right; *n* = 6) participants, for 4 select ROIs (values normalized to within-subject mean cerebellar gray). Error bars represent standard error of the mean. Data for *n* = 16 participants with 60 min of data are similar.

Average time-activity curves (TACs) and time-varying SUVRs for each diagnostic group are shown in Fig 1. TACs are shown for cerebellum and for 4 other brain regions: frontal cortex, entorhinal cortex, parahippocampal gyrus, and fusiform gyrus. All brain regions demonstrated peak uptake of the tracer shortly after injection, followed by washout that was substantially faster in cerebellum than in other brain regions. Both HC and AD participants showed THK5351 uptake above reference levels in the cortical regions tested. In HC, this uptake was low, with subtly increased binding in temporal relative to frontal regions. In AD, uptake in these regions was substantially higher than in HC, with temporal binding exceeding frontal binding within AD participants. Relatively low retention compared to other regions (indicative of little to no expected pathology) in cerebellar gray matter confirms that this is a suitable reference region for THK5351 uptake analysis. In addition, these curves appear to show steady-state kinetics by about 40 min through the entire experiment for all ROIs. This is confirmed in the time-varying SUVRs (ROI/cerebellar gray), which indicate that SUVRs in the cortical regions are stable after about 40 min.

### Tracer measurement stability: 60 min vs. 90 min data

**Fig 2:**
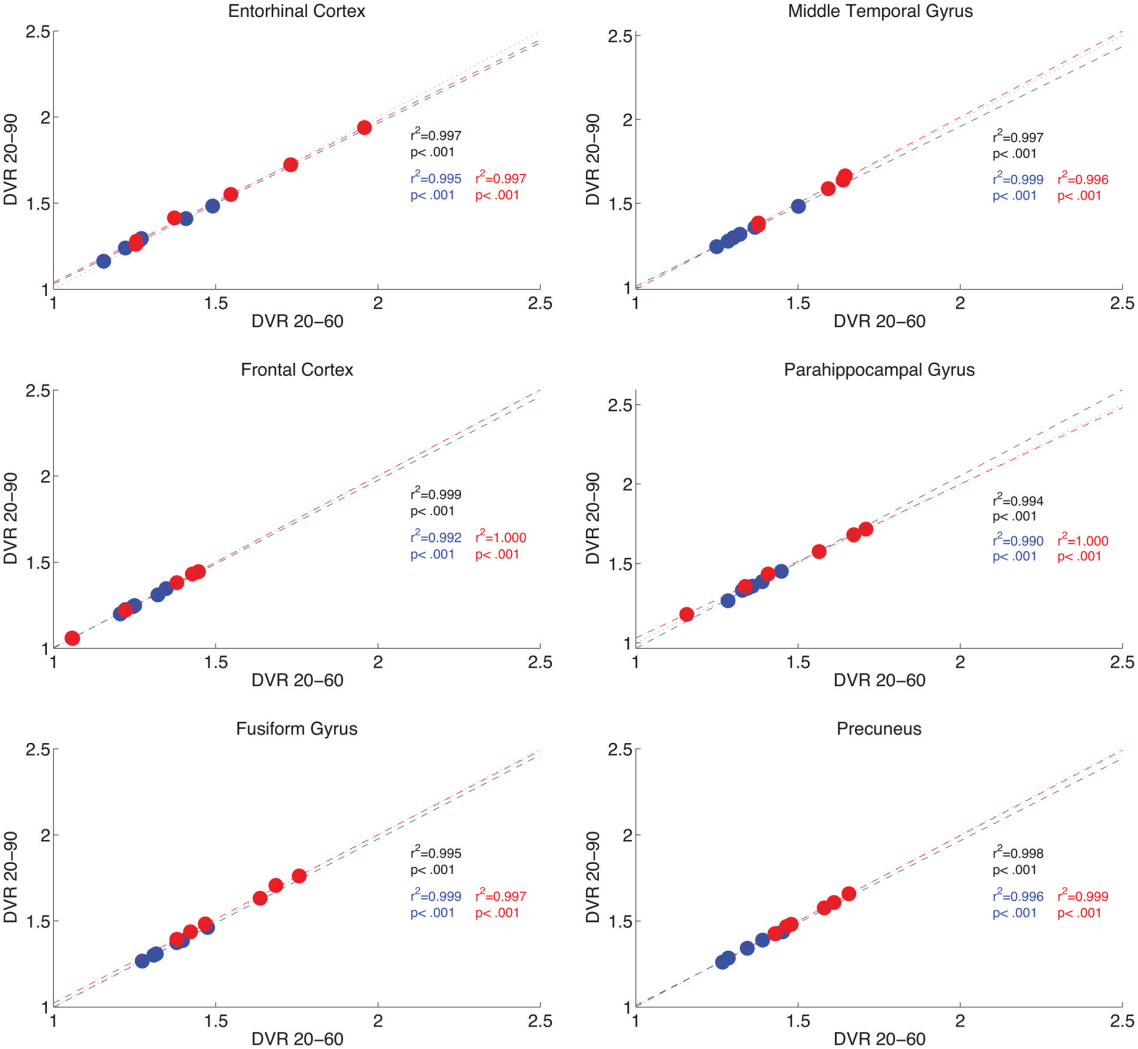
60 min DVRs correlate with 90 min DVRs. For *n* = 12 participants (HC in blue, AD in red) with 90 min of data, for 6 select ROIs, scatter plots illustrate relations between 20–60 min DVR data (x-axes) and 20–90 min DVR data (y-axes). Correlation results are included for all participants (black), and reported separately for AD (red) and HC participants (blue). Lines of best fit are presented in each group color (dashed), with identity represented by a dotted black line.

Because not all participants were able to complete a full 90 min dynamic PET acquisition, we investigated the relationship between 90 and 60 min data acquisitions, by analyzing the cases with 90 min of data using both 90 and 60 min pipelines. Results show a strong correlation of regional uptake values between shorter (20–60 min) or longer (20–90 min) time windows. For the 12 participants for whom 90 min of data are available, results are shown in Fig 2.

### DVR and SUVR become stable at later time frames

**Fig 3:**
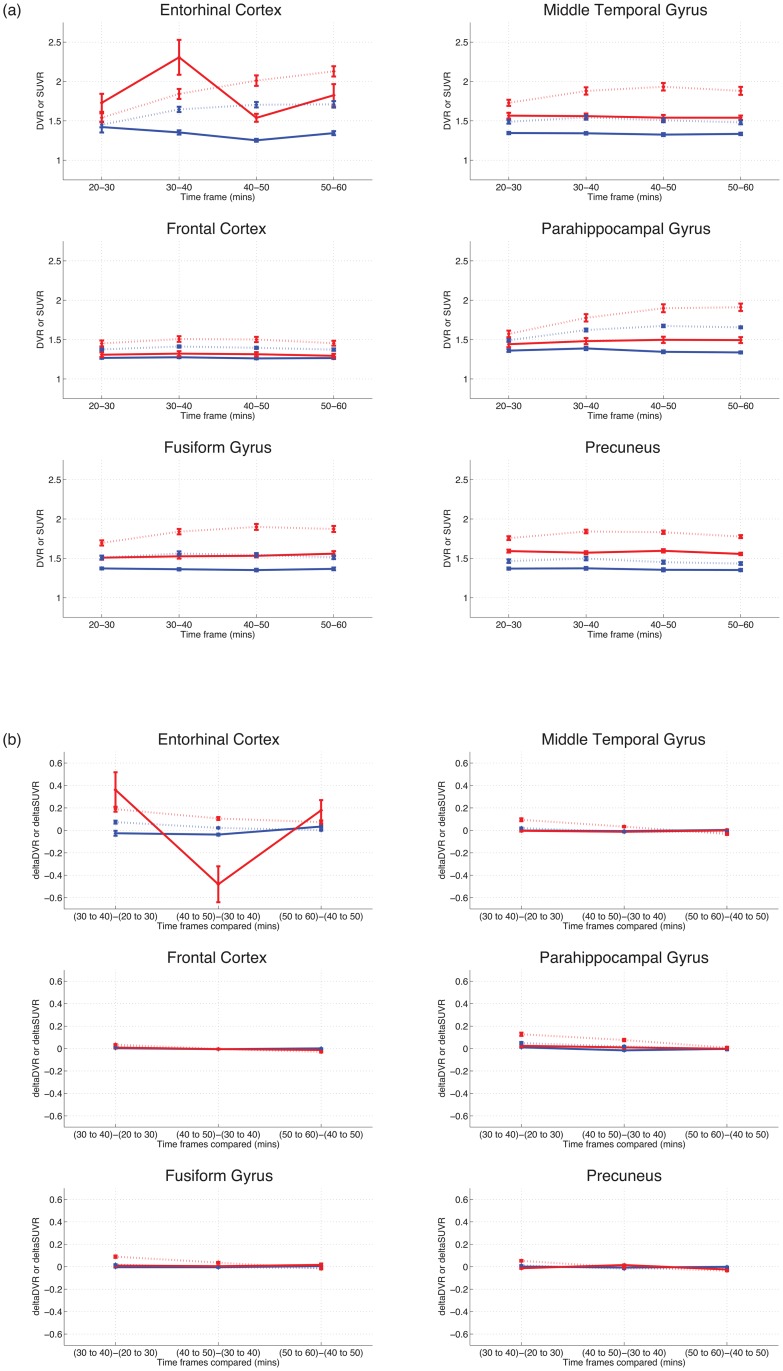
DVR and SUVR values at different time intervals. Results show that values are relatively stable after 40 min. Figures are for 20–60 min, with HC (*n* = 6) in blue, AD in red (*n* = 10). (a) Mean DVRs (solid lines) and SUVRs (dashed lines) over 4 10 min time windows, for 6 select ROIs. (b) Change in mean DVR (solid lines) and SUVR (dashed lines) values, over 4 10 min time windows, for 6 select ROIs.

We next examined DVR and SUVR stability across time, looking at signal and change in signal over time for both methods; results are shown in Fig 3. Both DVR and SUVR values show minimal change over time after approximately 40 min. Entorhinal results may be less stable due to the difficulty of segmenting this region correctly using automated imaging procedures, particularly in AD patients, and is discussed further below.

### Relationships between DVR and SUVR over time

**Fig 4:**
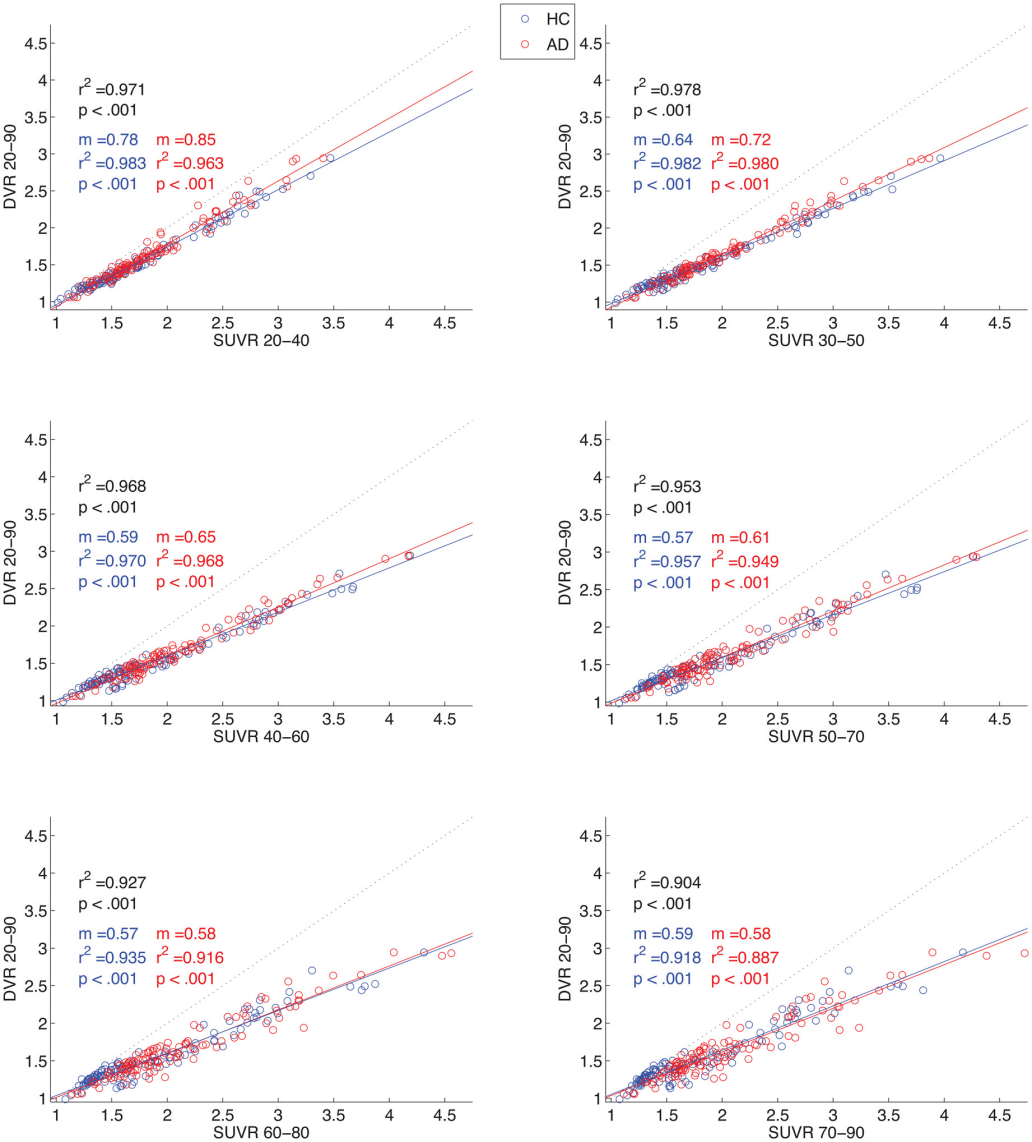
DVR vs. SUVR over time. Scatter plots and correlation results (*r*^*2*^, *p*) over six 20 min intervals. Correlation results are included for all participants (black), and reported separately for AD (red) and HC participants (blue). Slope (m) values are presented for each sub-group as well (DVR divided by SUVR; lower value indicates higher SUVR value relative to DVR). Lines of best fit are presented in each group color, with identity represented by a dotted black line.

The correlations between DVR values (20–90 min) and SUVR values over different time frames can be seen in Fig 4. The earliest 3 intervals (20–40 min, 30–50 min, and 40–60 min) demonstrate the highest correlation values with DVR 20–90 min, regardless of group status. Therefore, SUVR 20–40, 30–50, and 40–60 min appear to be the most consistently correlated with DVR across groups, while SUVR 40–60 (and later) appear to be the most stable (e.g., Fig 3). For this reason, SUVR 40–60 min was used in further group analyses.

### Description of SUVR 40–60 images in HC and AD

Brain SUVR images (40–60 min data, cerebellar gray matter reference) of THK5351 uptake are displayed in Fig 5. These mean and representative images illustrate that in certain brain regions, such as the basal ganglia, AD and HC participants accumulate high levels of THK5351 regardless of diagnostic group. However, within other regions, such as lateral temporal and parietal cortex, AD patients demonstrate greater uptake than HC. These findings were explored further with ROI-based statistical analyses.

**Fig 5:**
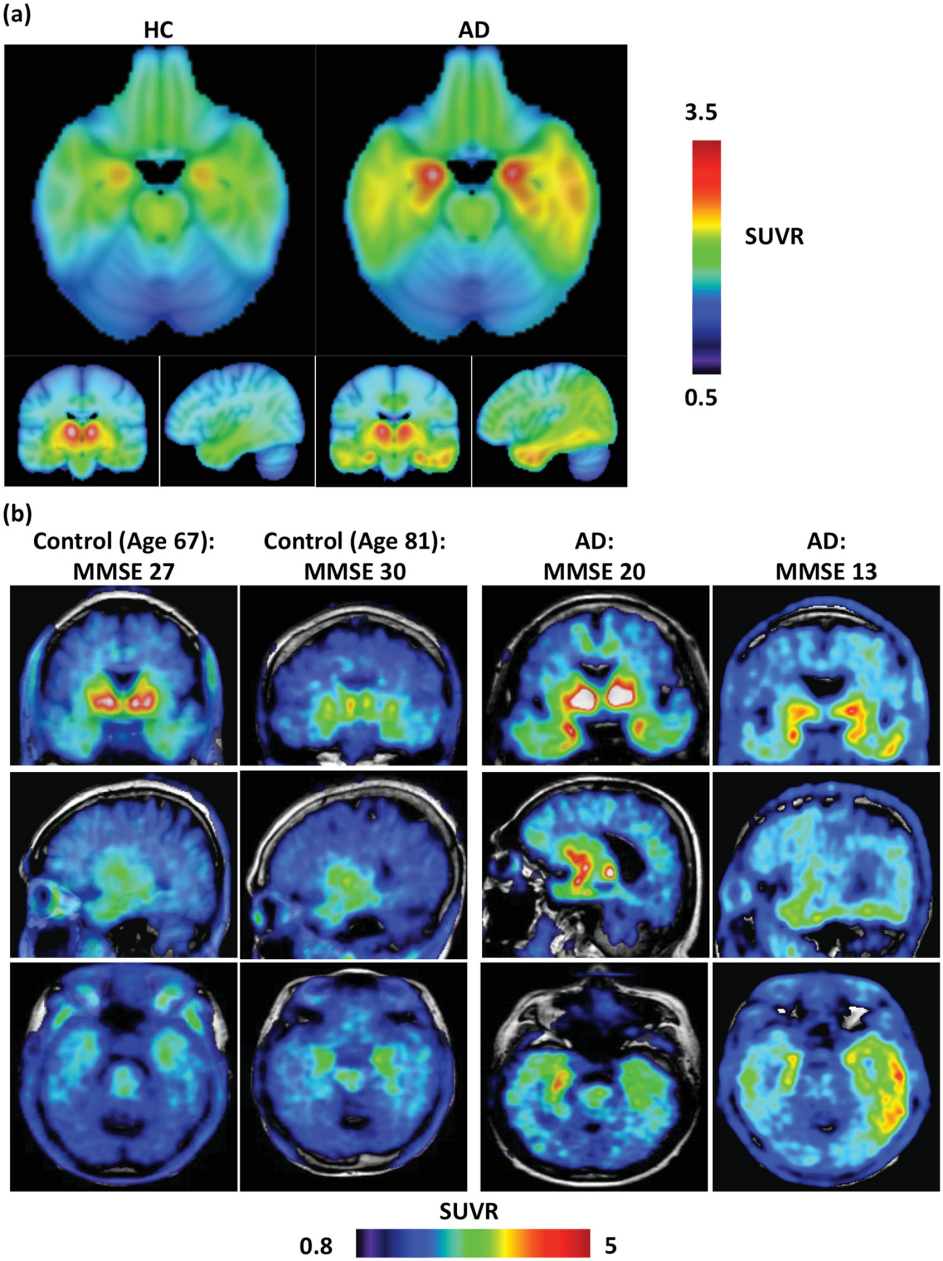
Average and representative THK5351 SUVR 40–60 min PET images overlaid on MRI images. (a) Average images for 6 HC (L) and 10 AD (R) participants, using a cerebellar gray matter reference region. Neurological convention: right hemisphere on right. (b) Representative images of 4 participants. SUVR range differs between average and single-subject images to illustrate full dynamic range of SUVR binding across participants.

### Group differences in 40–60 min SUVR

**Fig 6:**
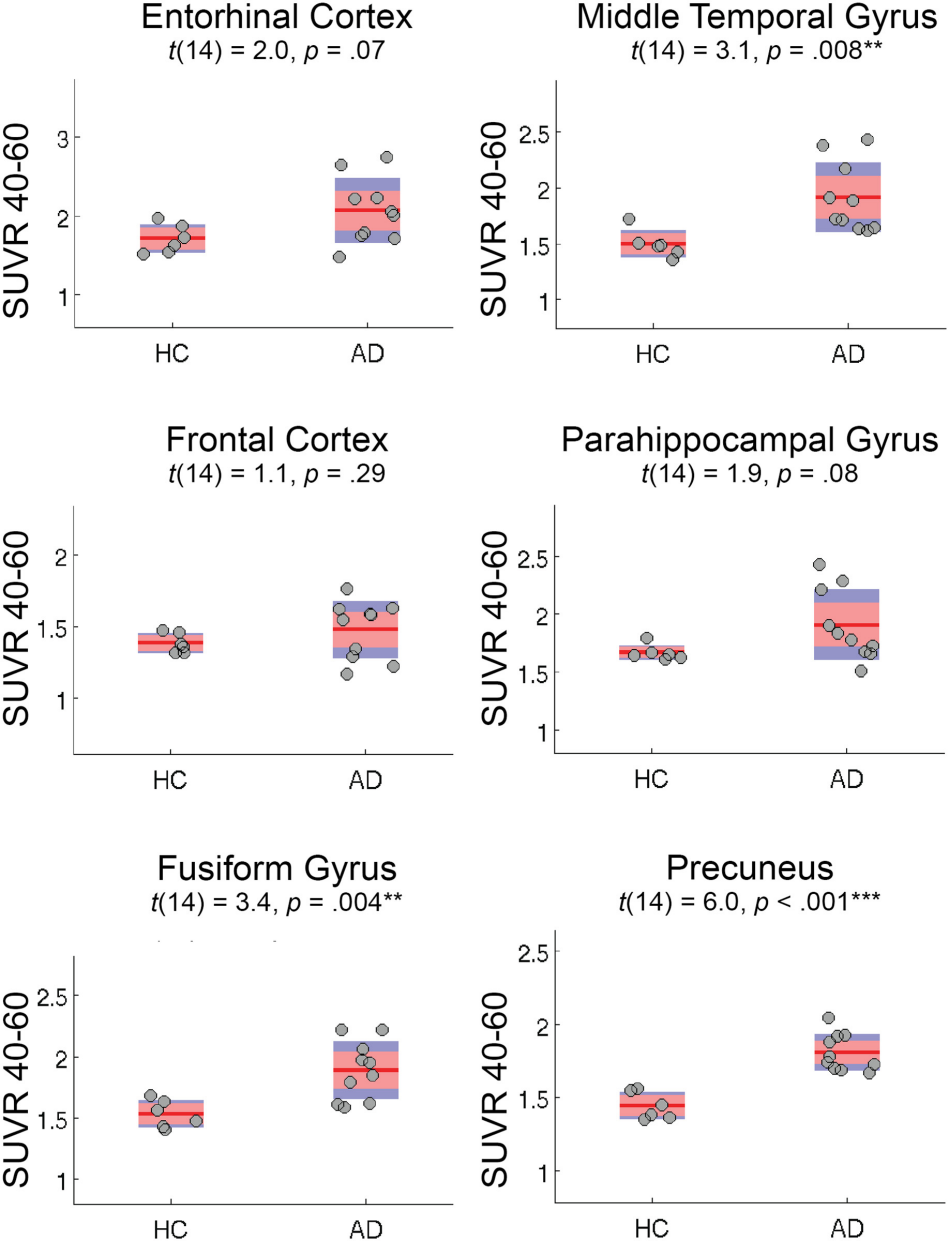
Group differences in THK5351 SUVR 40–60 min data. For 6 select ROIs, red bars represent 1.96 SEM (95% C.I.), and blue bars represent 1 SD, for 6 HC and 10 AD participants. Similar results were obtained when examining *n* = 12 participants with 90 min data sets.

Fig 6 shows SUVR 40–60 min differences between HC and AD participants (*n* = 16) for 6 select ROIs. Table 2 shows the values for all ROIs examined. Generally, significant (*p* < .05) group differences existed in temporoparietal regions, while marginal differences existed in medial temporal lobe subregions. A significant difference was also noted in the eroded white matter mask, which we expand upon in the discussion. High values of SUVR 40–60 min signal, but no group differences, were noted in basal ganglia regions. However, analyses of group differences in tracer uptake should be interpreted as exploratory, given our limited sample size.

**Table 2:** ROI values for group differences in SUVR 40–60 min.

ROI	HC mean	AD mean	T value‡	Significance
Anterior cingulate	1.88	1.89	0.1	*p* = .92
Brainstem	2.04	1.95	-1.2	*p* = .26
Caudate nucleus	2.73	2.44	-1.7	*p* = .12
Eroded white matter	1.6	1.82	3.2	*p* = .006*
Entorhinal cortex	1.71	2.06	2.0	*p* = .07
Frontal cortex	1.39	1.48	1.1	*p* = .29
Fusiform gyrus	1.53	1.89	3.4	*p* = .004*
Hippocampus	2.26	2.48	1.6	*p* = .14
Inferior temporal cortex	1.5	1.95	2.8	*p* = .01*
Lingual gyrus	1.29	1.51	3.6	*p* = .003*
Middle temporal gyrus	1.5	1.91	3.1	*p* = .008*
Occipital cortex	1.17	1.41	2.4	*p* = .03*
Pallidum	3.58	3.66	0.4	*p* = .68
Parahippocampal gyrus	1.67	1.9	1.9	*p* = .08
Parietal cortex	1.35	1.65	6.3	*p* < .001†
Posterior cingulate	1.65	1.93	4.3	*p* < .001†
Precuneus	1.44	1.81	6.0	*p* < .001†
Putamen	2.92	2.96	0.2	*p* = .83
Thalamus	2.94	2.83	-0.9	*p* = .41

* *p* < .05.

^†^ *p* < .001.

^‡^ All *t* results with df = 14.

## Discussion

In the current study, we explored tissue binding for the novel tau tracer THK5351 with multiple measures, in order to assess feasibility for human studies with a clinical focus. We primarily performed and examined relations between regional THK5351 DVR and SUVR calculations, seeking to describe associations between these measures, and searching for a 20 min SUVR that was both stable over time and comparable to DVR. We further explored diagnostic group differences in this optimal 20 min SUVR. And as 4 AD participants were unable to complete the final 30 min of a 90 min scan window, we examined within-measure associations between 60 min and 90 min data as well.

We found that analysis of 40–60 min THK5351 SUVR data provided optimal feasibility in several analyses, by showing good stability, comparison with DVR, and diagnostic group differences. Measurements from later time frames of data become more stable by reaching minimal to no change over time, at approximately 30–40 min. However, the earliest SUVR time frames were most strongly correlated with DVR results, although all time frames had high SUVR-DVR correlations. SUVR 40–60 min, within this “sweet spot” time window, showed expected group differences between AD and HC participants, suggesting it is a more optimal time frame to examine retention of this tracer in human subjects.

In addition, we examined group differences (AD > HC) in the ROIs measured. A pattern of increased uptake in neocortical regions in AD participants relative to controls, particularly in temporal cortex, was observed. Differences were marginal but not significant in the medial temporal lobe (hippocampus, entorhinal cortex), which is in agreement with neuropathology showing tau deposition in normal subjects in this region, and with the previous THK5105 compound [21], which do not find significant differences in tau accumulation in these regions. Our results are also in agreement with the smaller pathology and PET sample described recently by Harada et al. [22] using this same tracer. It must be noted that analyses of group differences in tracer uptake in the current are exploratory, given our limited sample size, and the primary methodological aims of this manuscript. However, the current results greatly extend upon previous understanding of the kinetics and choice of analysis time frame for the THK5351 tracer than any previous study. Smaller but significant differences were observed in eroded white matter and occipital cortex (Table 1). The finding of increased binding in the eroded white matter mask was surprising, as it is not consistent with the known distribution of tau pathology in AD [4]. This imaging finding could be related to mask selection, as partial volume effects could affect uptake measured from gray and white matter ROIs. Alternatively, this potential non-specific tracer retention in the subcortical white matter may represent binding of THK5351 to β-sheet structures in myelin [23], as this finding has been observed in previous THK series tracer data as well [21], though to a considerably greater extent. This pattern of results is consistent with previous findings in the area of AD vs. HC tau pathological differences [4–5, 22], and supports the use of this tracer as a biomarker for *in vivo* tau pathology and for examining regional differences in tau accumulation between groups of participants. Finally, in addition to the diagnostic differences tested here (e.g. HC vs. AD), differences in global brain amyloid burden (e.g., assessed using PiB PET) could also be associated with group differences we found in patterns of THK5351 uptake; however such analyses are beyond the scope of the current manuscript.

SUVR approaches are desirable in many clinical and research settings, relative to DVR [15]. SUVR has the advantage of brief, and therefore more feasible, scan data collection in older and demented adult participants, and SUVR calculations are simpler to perform than DVR analyses. Conversely, depending on the tracer and its pharmacokinetics, SUVR can overestimate results relative to DVR, in a manner that increases with time from injection [19]. This also appears true with the current dataset, where overestimation of SUVR relative to DVR increases as later 20 min time windows are examined using SUVR (S2 Fig). In this study, we confirmed 40–60 min to be an optimal time window for SUVR analysis of THK5351 PET data, as it provided results that were highly correlated with semi-quantitative dynamic measures (DVR), and permitted detection of regional clinical group differences.

THK-series tracers have been shown to be useful tau PET tracers because they show good specificity for the tau protein, relatively good signal-to-noise ratio, and low off-target binding. THK5351 has here been shown to be a typical, though improved, member of this molecular family [22]. THK5351, relative to other THK compounds (such as THK5105 and THK5117), has less white matter binding, and an improved pharmacokinetic profile, which potentially enhanced our ability to detect group differences [21]. Limitations of the current study include a small sample size, the absence of arterial blood sampling data, and difficulty segmenting smaller regions of interest. For example, it should be noted that entorhinal cortex is difficult to segment correctly using automated procedures, particularly in AD patients. This can lead to less stable values (particularly for short time windows) for this small but important ROI (e.g., see Fig 3); difficulties with segmentation and patient motion may have produced the unstable findings in our data. However, the pattern of results suggests that the current findings will bear out in larger studies using this and related tracers.

In summary, we examined THK5351, a new PET tracer for measuring accumulation of pathological tau protein, and described and compared multiple analysis methods for the quantification of regional tracer accumulation. SUVR 40–60 min performed optimally when examining 20 min SUVR time windows; this appears to be a practical method for quantification of retention of this tracer. The current results not only begin to inform our understanding of the mechanisms of tau pathology in normal aging and neurodegenerative disease, but also contribute to the body of empirical and theoretical research on tau PET imaging tracers, particularly regarding the kinetics of THK5351. Further, given the usefulness of THK5351-PET as a biomarker of tau pathology in aging and disease, these results also offer great clinical potential. Future examination of THK5351 and related tracers will assist the identification and study of preclinical pathological tau accumulation in the living human brain, a principal goal of researchers of brain aging and neurodegenerative disease.

## Supporting Information

S1 Fig Comparison of DVR 20–90 min results with SRTM BP 90 min results. Data are for *n* = 12 participants with full 90 min datasets (HC in blue, AD in red), for all ROIs except cerebellar gray. Means for each subject group illustrated with crosses.
(DOCX)

S2 Fig Slope of DVR (20–90 min) vs. SUVR for 6 20 min time intervals. Data are for *n* = 12 participants with full 90 min datasets (HC in blue, AD in red), for all ROIs except cerebellar gray. SUVR overestimation (lower slope) increases with time.
(DOCX)

S1 Table Regions of interest (ROIs) used in current study. (DOCX)
