# Supplementary material for: Dynamic PET Measures of Tau Accumulation in Cognitively Normal Older Adults and Alzheimer’s Disease Patients Measured Using [18F] THK-5351
Source: PLoS One. 2016 Jun 29;11(6):e0158460. doi: 10.1371/journal.pone.0158460 (PMC4927104; doi:10.1371/journal.pone.0158460)
Supplement: S1 Fig — Data are for n = 12 participants with full 90 min datasets (HC in blue, AD in red), for all ROIs except cerebellar gray. Means for each subject group illustrated with crosses. (DOCX) [file pone.0158460.s001.docx]

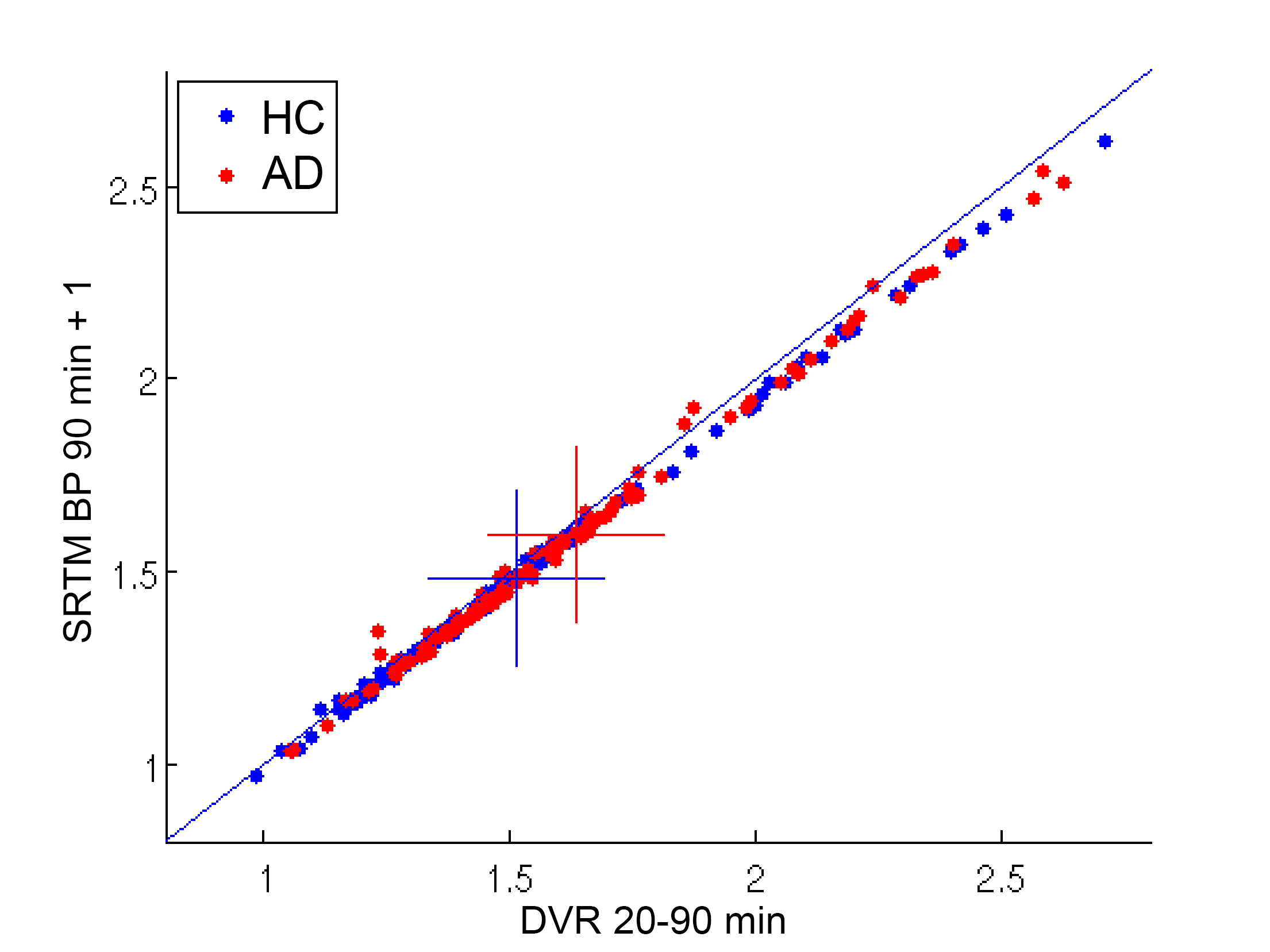


**S1 Figure.** **Comparison of DVR 20-90 min results with SRTM BP 90 min results**. Data are for *n* = 12 participants with full 90 min datasets (HC in blue, AD in red), for all ROIs except cerebellar gray. Means for each subject group illustrated with crosses.
