# Supplementary material for: Dynamic PET Measures of Tau Accumulation in Cognitively Normal Older Adults and Alzheimer’s Disease Patients Measured Using [18F] THK-5351
Source: PLoS One. 2016 Jun 29;11(6):e0158460. doi: 10.1371/journal.pone.0158460 (PMC4927104; doi:10.1371/journal.pone.0158460)
Supplement: S1 Table — (DOCX) [file pone.0158460.s003.docx]

**S1 Table. Regions of interest (ROIs) used in current study.**

| Temporal ROIs | Entorhinal cortex, Fusiform gyrus, Hippocampus, Inferior temporal gyrus, Lingual gyrus, Middle temporal gyrus, Parahippocampal gyrus |
| --- | --- |
| Other cortical ROIs | Anterior cingulate, Frontal lobe, Occipital lobe, Parietal lobe, Posterior cingulate, Precuneus |
| Subcortical ROIs | Brainstem, Caudate Nucleus, (eroded) Cerebral white matter, Pallidum, Putamen, Thalamus |
| Reference ROI | Cerebellar gray |
